# Potent Neutralization of Botulinum Neurotoxin/B by Synergistic Action of Antibodies Recognizing Protein and Ganglioside Receptor Binding Domain

**DOI:** 10.1371/journal.pone.0043845

**Published:** 2012-08-29

**Authors:** Changchun Chen, Shuhui Wang, Huajing Wang, Xiaoyan Mao, Tiancheng Zhang, Guanghui Ji, Xin Shi, Tian Xia, Weijia Lu, Dapeng Zhang, Jianxin Dai, Yajun Guo

**Affiliations:** 1 School of Pharmacy, The Center for Antibody Medicine of Ministry of Education, Shanghai Jiao Tong University, Shanghai, People's Republic of China; 2 International Joint Cancer Institute, The Second Military Medical University, Shanghai, People's Republic of China; 3 National Engineering Research Center for Antibody Medicine, State Key Laboratory of Antibody Medicine and Targeting Therapy and Shanghai Key Laboratory of Cell Engineering, Shanghai, People's Republic of China; 4 Lanzhou Institute of Biological Products, Lanzhou, Gansu, People's Republic of China; 5 PLA General Hospital Cancer Center, Beijing, People's Republic of China; Institute Pasteur, France

## Abstract

**Background:**

Botulinum neurotoxins (BoNTs), the causative agents for life-threatening human disease botulism, have been recognized as biological warfare agents. Monoclonal antibody (mAb) therapeutics hold considerable promise as BoNT therapeutics, but the potencies of mAbs against BoNTs are usually less than that of polyclonal antibodies (or oligoclonal antibodies). The confirmation of key epitopes with development of effective mAb is urgently needed.

**Methods and Findings:**

We selected 3 neutralizing mAbs which recognize different non-overlapping epitopes of BoNT/B from a panel of neutralizing antibodies against BoNT/B. By comparing the neutralizing effects among different combination groups, we found that 8E10, response to ganglioside receptor binding site, could synergy with 5G10 and 2F4, recognizing non-overlapping epitopes within Syt II binding sites. However, the combination of 5G10 with 2F4 blocking protein receptor binding sites did not achieve synergistical effects. Moreover, we found that the binding epitope of 8E10 was conserved among BoNT A, B, E, and F, which might cross-protect the challenge of different serotypes of BoNTs *in vivo*.

**Conclusions:**

The combination of two mAbs recognizing different receptors' binding domain in BoNTs has a synergistic effect. 8E10 is a potential universal partner for the synergistical combination with other mAb against protein receptor binding domain in BoNTs of other serotypes.

## Introduction

Botulinum neurotoxins (BoNTs) comprise a group of highly lethal toxins consisting of 7 serotypes (BoNT/A-G) produced by the anerobic bacteria, Clostridium botulinum [1], [2]. Four of the BoNT serotypes (A, B, E, and F) cause human botulism, a neuroparalytic disease which results from ingestion of pre-formed toxin present in contaminated food and from toxin produced *in vivo* from infected wounds [3]. Owing to their extreme potency and lethality, BoNTs are included in the list of category A select agents and toxins [4]. Each BoNT isoform is synthesized as a single polypeptide chain with a molecular mass of ∼150 kDa. The inactive precursor protein is cleaved either by clostridial or tissue proteases into a 50-kDa light chain (LC) and a 100 kDa heavy chain (HC) linked by an essential interchain disulfide bridge and by the belt, a loop from the HC that wraps around the LC [5]. The LCs act as zinc metallopeptidases, which solely hydrolyze one of three SNARE proteins depending on the serotype: BoNT A and E cleave synaptosomal-associated protein of 25 kDa (snap-25) and BoNT/B and F cleave the vesicle associated membrane protein (VAMP) [6], [7], [8], [9]. resulting in a blockade of neurotransmission and flaccid paralysis [10]. The heavy chain is divided into two functionally distinct regions: a C terminal binding domain (Hc) and a N terminal translocation domain (H_N_) [11]. The binding domain initially interacts with low affinity to a group of gangliosides on the presynaptic plasma membrane [12], after which it binds to a protein acceptor. Interestingly, the BoNTs' serotypes that exhibit highest sequence similarity share the same protein receptor, i.e., BoNT types A, E, and F bind SV2 [13], [14], [15], whereas BoNT types B bind SytI and II [16]. The existence of two classes of binding sites distinguished by different affinities and the discovery of protease-sensitive binding to neurons resulted in a double-receptor concept. In a first step complex polysialogangliosides accumulate BoNTs on the plasma membrane surface; and in a second step, protein receptors mediate their endocytosis.

Monoclonal antibodies (mAbs) have been intensely explored as inhibitors of the recognition step between BoNTs and their cellular receptors. [17] However, the two-receptor model makes it difficult to development of a mAb-based antitoxin for botulism. Single mAb recognizing only one epitope can hardly block the binding between BoNT and cell completely. It might be the reason why single mAb can only neutralize at most 10 to 100 times the 50% lethal dose (LD50) of toxin in mice [17]. Combination of two or three mAbs recognizing nonoverlapping epitopes can neutralize BoNT A very potently [18]. Although the binding areas of antibodies were mapped, however, the epitopes which these antibodies bind to are not finely defined. However, not all randomly paired mAbs have the potent synergistical neutralizing function. It is more difficult to select mAb pairs with desirable synergistical function from a panel of neutralizing mAbs whose epitopes are not clear. Because both the protein receptor and ganglioside receptor are essential for the entrance of BoNTs into neurons [19], we predicted that two monoclonal antibodies, which recognized protein and ganglioside receptor binding domain respectively, can neutralize synergistically against BoNTs.

In this study, we selected three neutralizing mAbs, which recognize different non-overlapping epitopes of BoNT/B, and compare the neutralizing effects among different antibody combinations. We found that 8E10, response to ganglioside receptor binding site, could collaborate with 5G10 and 2F4, recognizing non-overlapping epitopes within Syt II binding sites. However, the combination between two mAbs, 5G10 and 2F4, blocking protein receptor binding sites, did not achieve synergistical effects. We also found that the binding epitope of 8E10 was conserved among BoNT A, B, E and F. It could cross-protect the challenge of different serotypes of BoNTs *in vivo*. The results provide a potential universal partner for the synergistical combination with other mAb against protein receptor binding domain in BoNTs of other serotypes.

## Results

### 1. A panel of neutralizing antibodies specific for BoNT/B-Hc

**Figure 1:**
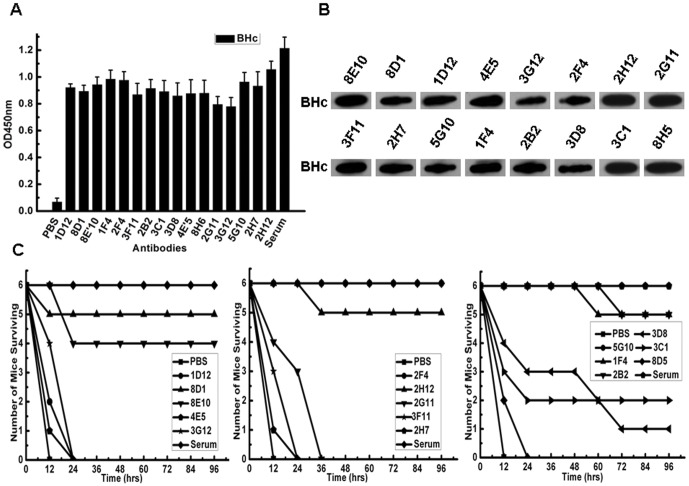
Characterization of mAbs specific for BoNT/B Hc. The binding activity of mAb was determined by ELISA (A) and Western blot analysis (B). Neutralizing mAbs were selected by *in vivo* neutralization assay(C). For the initial screening of the positive hybridoma that produced the neutralizing antibody, 200 µl of culture supernatant from hybridomas grown in a 24-well tissue culture flask for 3–5 days, was pre-incubated with 4 LD50 of BoNT/B for 1 hour at 37°C, and the reaction mixtures intraperitoneally injected to each of six mice. More than 4 survivals in four days were considered as positive. Serum of the immunized mice in the dilution of 1∶100 was set as control. Left: PBS, 1D12, 8D1, 8E10, 4E5, Serum. Middle: PBS, 2F4, 2H12, 2G11, 3F11, 2H7, Serum. Right: PBS, 5G10, 1F4, 2B2, 3D8, 3C1, 8H5, Serum. The error bars represented standard deviations.

To generate mAbs capable of neutralizing BoNT/B, gene encoding protective antigen Hc fragment of botulism neurotoxin serotype B (BoNT/B-Hc) was optimized and synthesized. The reconstructed Hc proteins were expressed in *E.coli* BL21 (DE3) in soluble form and purified for preparation of a toxoid immunogen. Sixteen hybridomas were obtained and screened for the production of anti-BoNT/B Hc mAbs, using ELISA with purified BoNT/B Hc (Fig. 1A). Cell culture medium corresponding to the positive wells was subjected to western blot assay for testing the binding specificity of these mAbs (Fig. 1B). The results showed that all of the positive hybridomas were able to bind to native or denatured BoNT/B Hc specifically. The supernatants of these clones were then tested for their ability to protect mice against 4 LD50 of BoNT/B for 4 days. Among them, 7 mAbs (8D1, 8E10, 1F4, 2F4, 5G10, 2H12 and 2B2) could neutralize BoNT/B *in vivo* (Fig. 1C).

### 2. Blocking activities of the neutralizing mAbs

**Figure 2:**
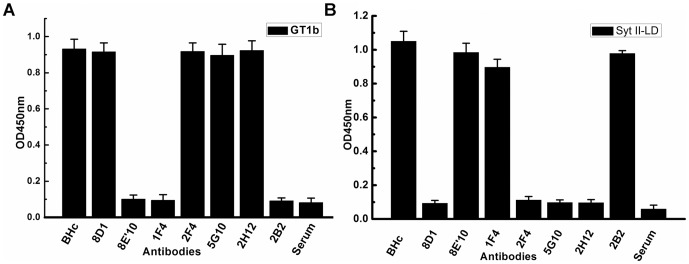
Binding domains recognized by neutralizing mAbs. Plates were coated with GT1b (A) or recombinant syt II-LD (B) respectively. HRP-conjugated BoNT/B Hc was pre-incubated with neutralizing mAbs at molar ratio of 1∶100. Bound BoNT/B Hc was detected by adding TMB as a substrate.

The first step in BoNT attachment to nerve membranes starts with C-terminus of Hc binding to gangliosides of neuron membranes. Ganglioside binding could either bring the toxin and the protein receptor into close proximity or cause a conformational change at the protein receptor binding site to enhance interactions [2]. To identify binding domain which was blocked by the seven neutralizing mAbs, we used ELISA-based analysis of GT1b or SytII binding with BoNT/B Hc pre-incubated with or without mAbs. The results indicated that mAb 8E10, 1F4 and 2B2 blocked the interaction between BoNT/B Hc and GT1b (Fig. 2A), whereas 8D1, 2F4, 5G10 and 2H12 interrupted the binding between BoNT/B Hc and Syt II (Fig. 2B).

To further investigate if the epitopes recognized by the antibodies were overlapped, we tested mAbs using competitive binding ELISAs. The optimal dilution of each HRP-conjugated mAb was determined by testing serial two-fold dilution in BoNT B Hc-coated ELISA plates and selecting this as the working dilution to generate an optical density of around 1.5. The selected working dilution was 1/8000 for mAbs 2B2 and 2F4, 1/16000 for mAbs 8D1, 1F4, 8E10, 5G10 and 1/4000 for 1F4, 2H12. The results of competitive ELISAs were reported in Table 1.

**Table 1:** Results of competition binding ELISAs for MAb epitope studies.

	unlabelled mAbs	HRP-conjugated mAbs						
		8E10-HRP	1F4-HRP	2B2-HRP	5G10-HRP	2H12-HRP	2F4-HRP	8D1-HRP
Group 1	8E10	9	7	8	90	92	90	92
	1F4	8	5	7	88	91	91	93
	2B2	6	9	8	92	88	95	93
Group 2	5G10	91	90	85	5	6	78	80
	2H12	87	90	89	7	4	81	79
Group 3	2F4	90	87	91	82	79	3	5
	8D1	88	93	87	80	78	6	4
	PBS	100	100	100	100	100	100	100

Results were expressed as percent binding of HRP-conjugated mAbs. The amount of binding obtained in the absence of unlabeled antibody was set at 100% for each HRP conjugated mAb. First column indicates the group assignments of mAbs based on competition binding assay. mAbs binding to overlapping epitopes are grouped together, while non-competing mAbs are grouped individually.

HRP-conjugated 8E10 inhibited the binding of 1F4 or 2B2 to the antigen and vice versa, showing that 8E10, 1F4 and 2B2 recognized overlapping epitopes. HRP-conjugated 5G10 inhibited the binding of 2H12 to the antigen and vice versa, indicating that 5G10 and 2H12 recognized overlapping epitopes. The same competition was also observed between 2F4 and 8D1. MAbs were thus categorized into 3 groups. MAbs binding to the same epitopes or overlapping epitopes were put into the same group (Table 2). Group 1 consisted of mAbs 8E10, 1F4 and 2B2, group 2 included mAbs 5G10 and 2H12 and group 3 included mAbs 2F4 and 8D1. Because the antigen binding affinity of the mAbs 8E10, 5G10 and 2F4 was higher than that of other 4 neutralizing mAbs (Table 1), they were selected for fine epitope mapping based on phage-display.

**Table 2:** Kinetic constants and isotypes of mAbs binding to BoNT/B-Hc.

mAb	*k*off (M-1 S-1)	*k*on (S-1)	*KD* (M)	Subtype
8E10	4.070E−4	1.838E+5	2.214E−9	IgG1/κ
2B2	5.941E−4	7.572E+4	7.854E−9	IgG1/κ
1F4	2.674E−4	4.605E+4	5.806E−9	IgG1/κ
5G10	4.146E−5	1.374E+4	2.975E−9	IgG1/κ
2H12	9.390E−3	6.631E+5	1.301E−8	IgG1/κ
2F4	1.085E−4	5.425E+4	2.000E−9	IgG1/κ
8D1	2.335E−3	2.468E+4	9.460−8	IgG1/κ

### 3. Epitope mapping of mAb 8E10, 5G10 and 2F4

**Figure 3:**
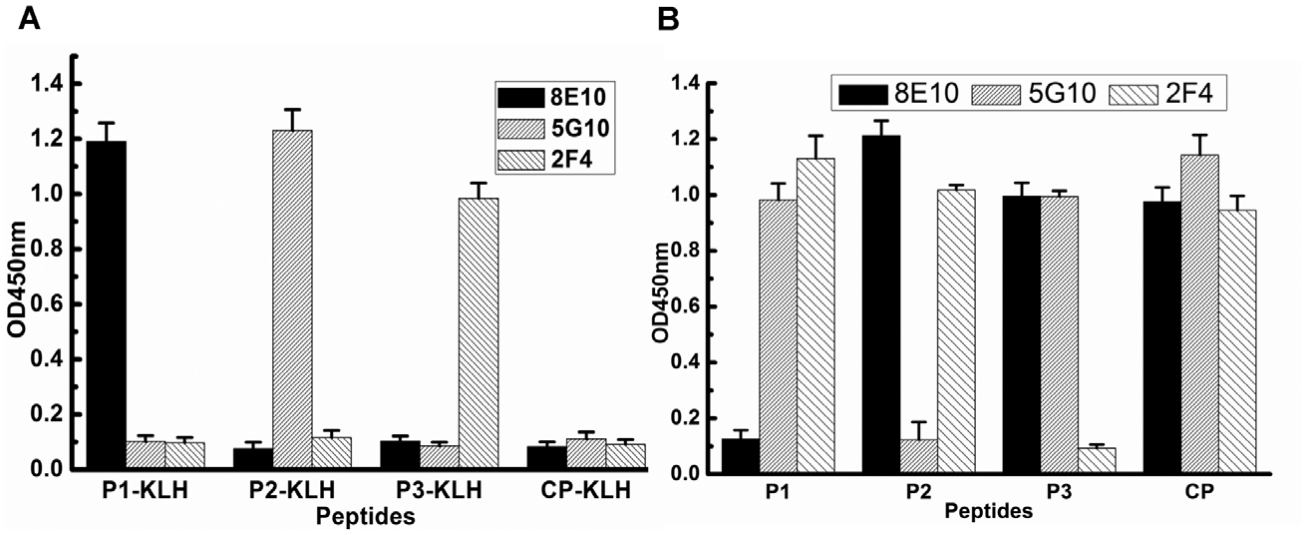
Reactivity of synthetic epitope peptides with neutralizing mAbs. Binding of synthetic peptide with mAb. Peptides P1, P2, P3, containing the motifs recognized by 8E10, 5G10 and 2F4 respectively, were synthesized and conjugated with KLH, which were indicated as P1-, P2-, P3-KLH. The binding abilities of mAbs were detected by ELISA. A non-associated peptide was set as control peptide (CP), and conjugated with KLH as CP-KLH. (B) Competitive inhibition of BoNT/B Hc binding with mAbs by synthetic peptides. CP-KLH: synthetic peptide THPHLPRALMRS was coupled to KLH (bearer protein) to generate CP-KLH; P1-KLH: synthetic peptide YFCISKWYLKEV was coupled to KLH (bearer protein) to generate P1-KLH. P2-KLH: synthetic peptide PISDSDEFYNTI was coupled to KLH (bearer protein) to generate P2-KLH; P3-KLH: synthetic peptide IKLKKDSPVGEI was coupled to KLH(bearer protein) to generate P3-KLH.

After three successive rounds of panning on 8E10, 5G10 and 2F4 respectively, we randomly selected and purified 100 positive phages. DNA sequence analysis of the phage-encoded peptides revealed 4–6 amino-acid consensus sequences, which was aligned with the sequence of BoNT/B displayed in Table 3. All phages selected by 8E10 have a common motif of SXWY. Alignment of this sequence on the sequence of BoNT/B provides a best fit with 1259SKWY1262. 5G10 and 2F4 bind to SDXFY and KXSP, which correspond exactly to residues 1201SDEFY1205 and 1114KDSP1117 of BoNT/B respectively. The peptides containing 1259SKWY1262, 1201SDEFY1205 and 1114KDSP1117 motif in the BoNT/B were synthesized and denoted as P1, P2 and P3 respectively. The ELISA assay results showed that mAb 8E10, 5G10 and 2F4 bound to KLH-conjugated P1, P2 and P3 peptides (P1, P2, P3-KLH) specifically since these mAbs did not cross-react with the other peptides recognized by the other two mAbs and control peptide-KLH (CP-KLH)(Fig. 3A). The capability of the synthesized peptides to block the binding of mAb 8E10, 5G10 and 2F4 to BoNT/B Hc was also determined. The results revealed that peptide P1, P2, P3 effectively inhibited the binding of mAb 8E10, 5G10, 2F4 to BoNT/B Hc respectively. In contrast, control peptide had not inhibitory effect on their interaction (Fig. 3B). These results further demonstrated that the motifs listed above were the epitopes of 8E10, 5G10 and 2F4 respectively.

**Table 3:** Alignment of phage-displayed peptide sequences selected by anti-BoNT/B neutralizing antibodies.

Phage clone	Phage-displayed peptide sequence	Absolute and relative frequency
**5G10 selected**		
5G10-A1	**--**AGQ**SD**S**YY**LYHP**--**	**12 (24%)**
5G10-G4	**--**SF**AD**Y**YY**QYSPR**--**	**4 (8%)**
5G10-C10	-**--**T**SD**E**WY**NLHFLR	**6 (12%)**
5G10-F6	**-----**TYK**SD**H**FY**HWQP**--**	**20 (40%)**
5G10-D1	**-----**TPT**SD**R**FF**NLWP**--**	**8 (16%)**
Consensus	**--SD**X**FY--**	
BoNT/B	**-^1201^SD**E**FY^1205^-**	
**8E10 selected**		
8E10-B9	**--**RTMKSM**SDWY**QK**--**	**3 (4.7%)**
8E10-D6	**---**TNSD**SEWF**GHYM**--**	**4 (6.3%)**
8E10-E3	**--**TKDAE**SRWY**HAF**--**	**24 (37.5%)**
8E10-F2	**---**SV**TDYF**NLTILQ**-**	**5 (7.8%)**
8E10-F10	**--**FS**TRWY**ESWLNP	**12 (18.8%)**
8E10-H1	**--**SMLG**SEYY**MMVM**--**	**16 (25%)**
Consensus	**---SXWY---**	
BoNT/B	**--^1259^SKWY^1262^---**	
**2F4 selected**		
2F4-A9	**---**MN**RDSP**EHIVAL**--**	**3 (5.9%)**
2F4-C11	**--**EALT**KHTF**TQLV**---**	**7 (13.7%)**
2F4-E8	**--**TMI**KHSET**FNQL**---**	**9 (17.6%)**
2F4-2A1	**--**IPR**KESP**YQRIW**--**	**21 (41.2%)**
2F4-2G4	**---**M**RQCP**KEHIVAL**-**	**11 (21.6%)**
Consensus	**---KXSP--**	
BoNT/B	**--^1114^KDSP^1117^--**	

**Phage-displayed consensus amino acids are shown in boldface.**

**Figure 4:**
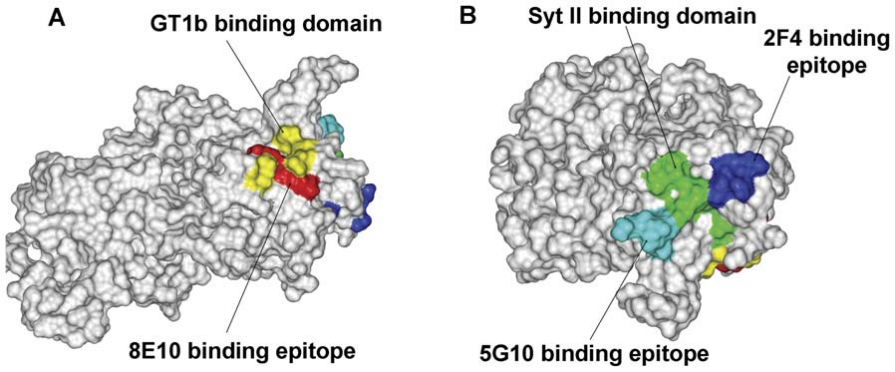
Molecular model overlay of neutralizing epitopes within the BoNT/B Hc binding domain. The model was established using the software Discovery Studio 2.0 (Accelrys, San Diego, CA) based on the crystal structure of BoNT/B Hc (PDB 1F31) from the Protein Data Bank. BoNT/B Hc is shown in a surface representation. (A) The residues reported as GT1b-binding site are colored yellow (*Nat. Struct. Biol. 1998*), and the residues recognized by 8E10 are colored red. (B) The SytII-binding site residues are colored green, and the residues of 5G10 and 2F4 are indicated in cyan and blue.

BoNT/B Hc has been crystalized in complex with trisaccharide sialyllactose (a mimic of GT1b) (PDB 1F31) and with SytII peptide (PDB 2NM1). The crystal structure reveals two distinguished domains which bind to GT1b and SytII respectively. Therefore, it is possible to compare the binding site of protein and ganglioside receptor to BoNT/B Hc with the localization of the mAb 8E10, 5G10 and 2F4 epitope identified in this study. The illustration of structure of BoNT/B Hc is showed in Fig. 4. The epitopes of mAbs were colored and overlaid on the three-dimensional structure of BoNT/B. The epitope of 8E10 (colored in red) is just located inside the GT1b-binding domain (colored in yellow) (Fig. 4 A). The residues “1114KDSP1117” recognized by 2F4 (Fig. 4B, blue) cover part of the Syt II binding pocket formed by residues K1114, S1116, P1117, V1118, Y1183, E1191, K1192, F1194 and F1204 (Fig. 4B, green). The 5G10 epitope comprises 5 amino acids “1201SDEFY1205” (Fig. 4B, cyan), which overlaps another side of Syt II binding site formed by residues P1117, W1178, Y1181, F1194, A1196, P1197 and F1204 (Fig. 4B, green). However, the epitopes of 5G10 and 2F4 are not overlapping with each other. These results provide the structure basis of neutralizing functions of these mAbs. The mAb 8E10 neutralizes BoNT/B through blocking the interaction between BoNT/B Hc and its ganglioside receptor on neurons. 5G10 and 2F4 inhibit the binding of BoNT/B Hc with protein receptor SytII.

### 4. Binding inhibition assay *in vitro*

**Figure 5:**
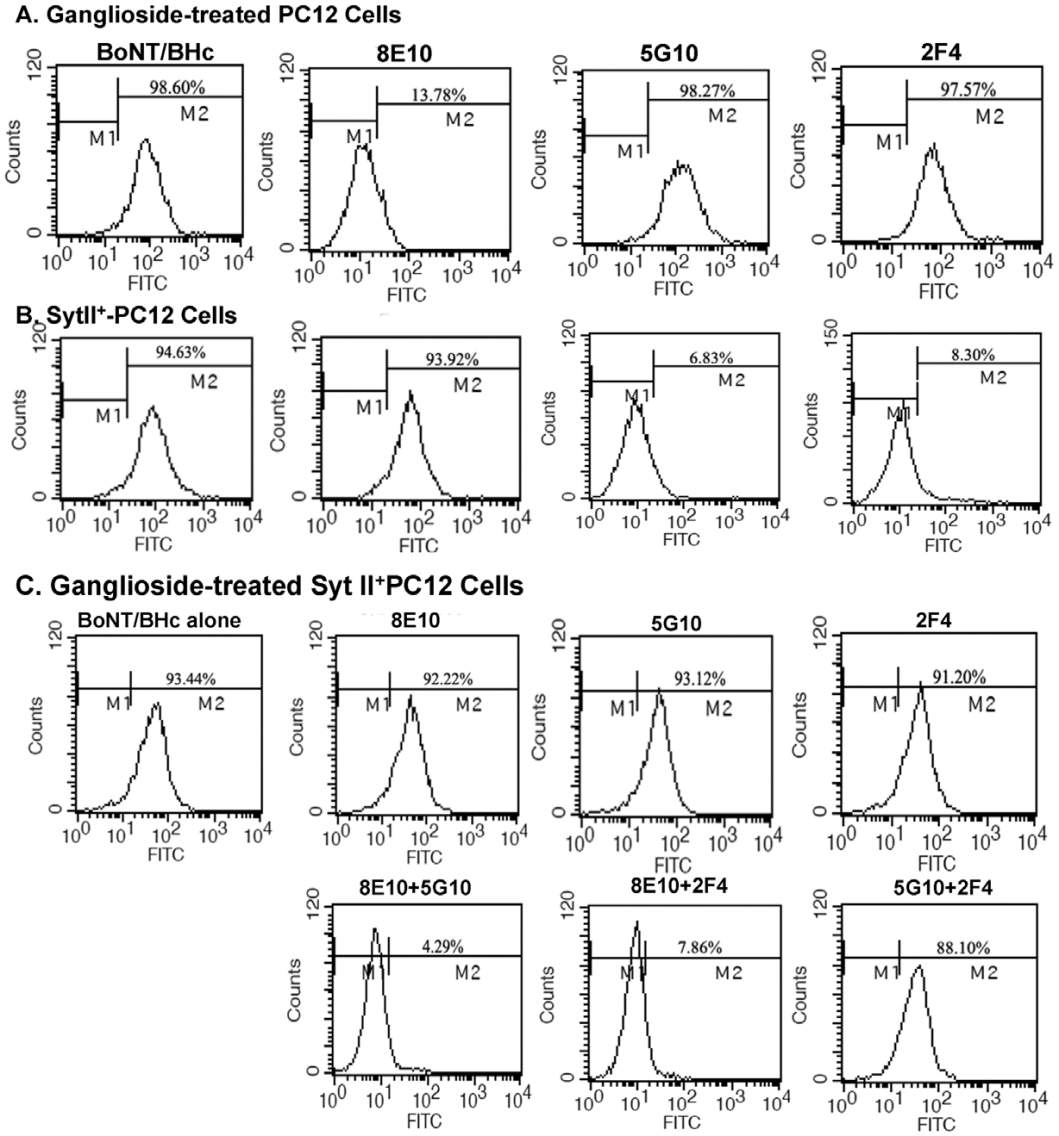
Inhibition of mAbs and pairs of mAbs to the interaction between BoNT/B and neurons by flow cytometer. The single or pairs of mAbs were mixed with FITC-BoNT/B Hc at a molar ratio of 1∶100 before staining PC12 cells treated as indicated. Inhibition of mAbs for neuron binding activity of BoNT/B was indicated by the flow cytometer. (A) ganglioside-treated cells and BoNT/B Hc with or without single mAb (8E10, 5G10 or 2F4) (B) the binding of SytII+ cells and BoNT/B Hc with or without single mAb (8E10, 5G10 or 2F4), (C) the binding of ganglioside-treated syt II+PC12 cells and BoNT/B Hc with or without single mAb (8E10, 5G10 or 2F4) or pairs of mAbs (8E10+5G10, 8E10+2F4, 5G10+2F4). For pairs of mAbs, the molar ratio between BoNT/B Hc and mAbs is 1∶50∶50.

In order to determine the toxin neutralization by IgG *in vitro*, we used SytII transfected and ganglioside treated PC12 cells, a neuroendocrine cell line of which wild type is lack of functional toxin receptors [20], to serve as a target cell model. Flow cytometry results showed that FITC-labeled recombinant BoNT/B Hc could bind to SytII transfected and ganglioside treated PC12. 8E10 could completely block the interaction between BoNT/B Hc and ganglioside treated PC12 (Fig. 5A). 5G10 and 2F4 could inhibit BoNT/B Hc to bind to the SytII transfected PC12 effectively (Fig. 5B). However, as to the SytII transfected and ganglioside treated PC12, none of the mAbs above could completely block its binding to BoNT/B Hc. In contrast with the single mAb, the combination of 8E10 with 5G10 or 2F4 could completely inhibit the binding of BoNT/B Hc to the double treated PC12, while the combination of 5G10 with 2F4 could not block the binding of PC12 to BoNT/B Hc (Fig. 5C). The results of confocal microscope showed that the combination of 5G10 and 2F4 could only decrease the fluorescence signal on the membrane of double treated PC12 similar to the effects of single antibody treatment. In contrast, the mixture of 8E10 and 5G10 or 2F4 abrogated the fluorescence signal from the cell membrane completely (Figure S1). These results demonstrated that mAbs, which recognized ganglioside or SytII binding domain respectively, had the potent to neutralize BoNT/B synergistically.

### 5. Protection of mice from BoNT/B toxicity using neutralizing mAbs

*In vivo* toxin neutralization was studied using a standard mouse protection assay. We incubated 100 µg mAbs with BoNT/B for 1 hour prior to intraperitoneal injection into BALB/c mice and the groups of mice with 6 animals for each group were used to test dose level. Mice were observed for morbidity and mortality over 30 days. Complete protection was observed with doses up to 20 LD50s for 8E10, and 40 LD50s for 5G10 and 2F4. Partial protection, as indicated by increased survival compared with antibody-free control mice, was afforded with higher doses (Table 4). At 80 LD50s, mice receiving 5G10 and 2F4 survived 72 hours, compared to 6 hours for the control mice. As combination of mAbs have demonstrated synergy in BoNT/A neutralization [18], the different pairs of mAb were studied at increasing doses of toxin to explore potent combination. We mixed 50 µg each of the mAbs (total of 100 µg) with different LD50s BoNT/B and tested the combination by intravenous injection. At 640 LD50s, only 1 of 6 mice receiving the pair of 5G10+2F4 survived whereas none of mice receiving two of the pairs of mAbs (8E10+5G10 or 8E10+2F4) died. 3 of 6 mice that received the pair of 8E10+5G10, and 2 of 6 mice that received the pair of 8E10+2F4 survived for 96 hours in the group challenged with 1,280 LD50s. In contrast, the control mice died in less than 3 hours. The survival rate of the group received 8E10+5G10+2F4 is the same as that of the group received the pair of 8E10+5G10. No mice survived in the group challenged with 2,560 LD50s (Data not shown).

**Table 4:** *In vivo* toxin neutralization by mAb alone, pairs or triplex of mAbs.

mAbs	BoNT/B LD_50_	Survival time (hours)	Survival (alive/tested)
Control	20	<12	0/6
5G10	20	96	6/6
8E10	20	96	6/6
2F4	20	96	6/6
Control	40	<8	0/6
5G10	40	96	6/6
8E10	40	72	0/6
2F4	40	96	6/6
Control	80	<6	0/6
5G10	80	72	0/6
8E10	80	48	0/6
2F4	80	72	0/6
Control	640	<4	0/6
5G10+2F4	640	60	1/6
8E10+5G10	640	96	6/6
8E10+2F4	640	96	6/6
8E10+2F4+5G10	640	96	6/6
Control	1280	<3	0/6
5G10+2F4	1280	24	0/6
8E10+5G10	1280	96	3/6
8E10+2F4	1280	96	2/6
8E10+2F4+5G10	1280	96	3/6

### 6. Neutralizing potency of mAb 8E10 against different serotypes of BoNTs

**Figure 6:**
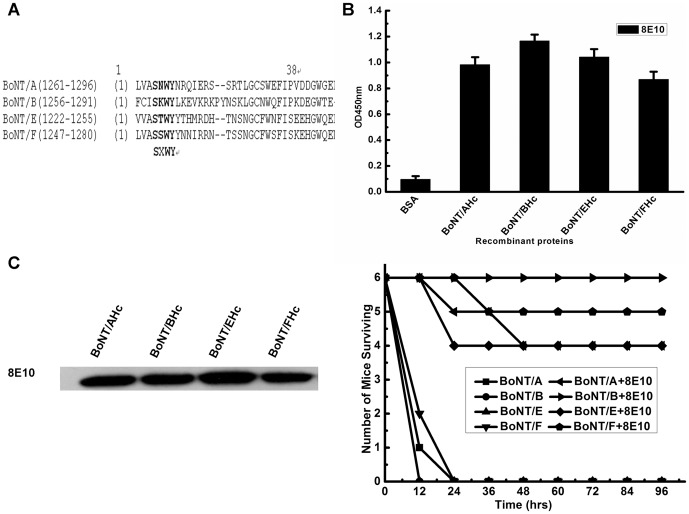
Cross-reactive and cross-neutralizing effects of 8E10. (A) Amino acid sequence alignment of the carboxyl-terminal region of BoNT (A, B, E, F). The residues, which are conservative among BoNTs/A, B, E and F are presented as bold letters. (B) ELISA of 8E10 to BoNT Hcs of different serotypes A, B, E and F. (C) BoNT Hcs (A, B, E, F) were separated by SDS-PAGE under reducing conditions and visualized by western blotting with 8E10. (D) Cross-neutralizing effects *in vivo*. 100 µg of 8E10 was pre-incubated with 20 LD50s of BoNT/A, B, E, F for 1 hour respectively. The mixtures were injected into mice, and the final death tally was determined 4 days after injection.

Given the binding epitope of 8E10 is located at the conservative motif of BoNT A, B, E, F (Fig. 6A), we wondered whether it could bind and neutralize across these serotypes. To evaluate this, we first expressed the Hc domain of BoNT A, E and F, and determined the binding kinetics of the 8E10 antibody to the BoNT/A, B, E, and F Hc by surface plasmon resonance (SPR) and showed the results in Table 5 and Figure S2. The results show that the binding affinity between 8E10 and BoNT/A or F is a little bit lower than the binding affinity between 8E10 and BoNT/E, whereas the affinity between 8E10 and BoNT/B is the highest. The fact that all four serotypes of BoNTs bind 8E10 with nanomolar efficiency demonstrates that all serotypes tested are of high affinity. The results of ELISA (Fig. 6B) and western blotting (Fig. 6C) showed that 8E10 could bind with native and denatured Hc of BoNT A, B, E, F, indicating that 8E10 recognized the conserved linear epitope among these BoNTs. In addition, we compared the potency of BoNT/A, B, E and F neutralization *in vivo* by mAbs 8E10. All of 6 mice challenged with 20 LD50s of BoNT/B were protected by 100 µg of 8E10. Same dosage of 8E10 protected 5/6 of mice challenged with 20 LD50s of BoNT/A or F, respectively and 4 of 6 mice also survived under the challenge of 20 LD50s of BoNT/E (Fig. 6D). In contrast, none of the mice survived longer than 24 hours after challenged with BoNTs without the injections of mAbs.

**Table 5:** Kinetic constants of mAb 8E10 binding to BoNT/A/B/E/F-Hc.

Serotype	*k*off (M-1 S-1)	*k*on (S-1)	*KD* (M)
BoNT A	1.077E−4	1.442E+4	7.468E−9
BoNT B	3.270E−5	1.261E+4	2.592E−9
BoNT E	6.209E−5	1.189E+4	5.223E−9
BoNT F	1.030E−4	1.339E+4	7.690E−9

## Discussion

For the last two decades, the relationship between the structure and function of BoNT has been studied intensively. With the complex structure of BoNT/B and its receptors, protein receptor Syt-II [21] and its ganglioside receptor GT1b [22], we can combine these data to provide the structure basis of the “double receptor” interaction proposed by Montecucco et al. [23]. Bio-functional assays also provided the evidence that both protein receptor SytII and ganglioside co-receptor were necessary for the infective process of BoNT/B [24]. This led us to the hypothesis that a combination comprised of a pair of neutralizing antibodies that bound to different receptors binding domains of the toxin would be more effective neutralizers than either member of the pair alone. We generated three mAbs, 8E10, 5G10 and 2F4 which could neutralize the challenge of 20 LD50 of BoNT/B *in vivo*. Finely epitope mapping revealed that 8E10 recognized ganglioside binding domain in BoNT/B, 5G10 and 2F4 bound with 2 non-overlapping epitopes surround the SytII binding domain respectively. In addition, we found that the combination between 8E10 (recognizing ganglioside binding domain) and 5G10 or 2F4 (recognizing protein receptor binding domain) could result in a more than 30–60 folds increase in potency compared with that of any the single mAb. However, for the combination of 5G10 and 2F4, which both blocked protein receptor binding sites by non-overlapping epitopes, no synergistic effect was observed. This indicates that double blocking the ganglioside and protein receptor binding domain simultaneously with two mAbs is helpful for the synergistic effects of the two antibodies' combination against BoNT/B.

The combinations of antibodies, which recognize nonoverlapping epitopes, synergistically cooperate in neutralization potency have been reported previously [18]. In that study, random combinations between 2 of the 3 neutralizing mAbs significantly prolonged the time to neuroparalysis compared with single mAbs. However, in our study, 2 mAbs, which bind to the same function domain of BoNT/B, had much less synergistic effects than those whose epitopes located in different function domains of BoNT/B. In addition, a BoNT/B-specific triplex antibody combination exhibited cooperative neutralizing effects to the toxin in vivo that were no better than those of the pairs of antibodies (Table 4). These findings are different from the conclusion of the previous report. Fine epitope mapping showed that the two mAbs (3D12 and S25) which bind the BoNT/A H_CC_ (C terminal of Hc) overlap the putative sialoganglioside binding site and cover a large portion of H_CC_. The other mAb C25 bound a conformational epitope that consisted of the sequence from the N- and C-terminal subdomains of BoNT/A Hc, which overlapped with a putative inositol phosphate binding site that may be important for attachment to the lipid membrane. This function domain is essential for the binding to anionic lipid in the environment of lipid raft, which is important for the translocation processing of the toxin. [25], [26]. Interestingly, the neutralizing potency of the pair of mAbs (C25+3D12), which bind to translocation domain and sialoganglioside binding domain respectively, is 10 times higher than that of S25+3D12, whose epitopes are located at the same function domain. This finding provides evidence that the synergetic effects of mAbs, which recognize different function domains of BoNT/A, are better than the effects of those recognizing the same function domain. Although we didn't observe the cooperative effects between the mAbs binding to SytII binding domain of BoNT/B, we did prove that the combination of two mAbs recognizing different receptors' binding domain in BoNT/B has a synergistic effect. Accordingly, the data indicate that the combination between 2 mAbs recognizing different function domains in BoNTs could be the general principle for the potent synergistic effect.

Neutralizing mAbs binding multiple serotypes of botulinum neurotoxin are rare have been reported previously [27]. The cross-reactive mAbs bound to a relatively conserved epitope at the tip of the BoNT H_N_. This is a functionally important epitope for intoxication, as mAb binding leads to potent BoNT neutralization. In this study, a neutralizing mAb 8E10 binding the conservative domain on Hc was reported. The structure of Hc from BoNT/B (PDB 2NM1) [21], BoNT/E (PDB 3FFZ) [28] and BoNT/F (PDB 3FUQ) [15] supports the view that the Hc fold is highly conserved. GT1b binds on a cleft formed by W1266 and Y1267 on one face and E1203, H1253, and F1252 on the other for BoNT/A [29]. The structure of Hc from BoNT/B in complex with the trisaccharide sialyllactose, a mimic of GT1b (PDB 1F31), displays a similar binding cavity with corresponding residues W1261, Y1262, and H1240 [22]. In addition, the crystal structure of Hc from BoNT/F displays a ganglioside-binding pocket with corresponding residues W1250, Y1251, and H1241 [15]. Amino acid sequence alignment shows that S1264, W1266 and Y1267, conserved among all BoNT serotypes [30], constitute key residues of a lactose-binding motif (H. . .SXWY. . .G) that contribute to the crevice binding with GT1b. The implication is that the GT1b-binding pocket for all these BoNTs is similar. We found in this study that the motif 1259SKWY1262 is the recognizing epitope of 8E10, which could also cross react with the Hc of BoNT A/B/E/F (Fig. 6). This conserved epitope may partially explain how 8E10 could cross-protect the challenge of different serotype of BoNTs *in vivo*. Although its synergistic effect with the mAbs blocking protein receptor binding domain in BoNT A, E or F has not been detected, we can predict that 8E10 could act synergistically with the mAbs recognizing protein receptor binding domain against BoNT A, E or F. This study provides a potential universal partner for the synergistical combination with other mAb against protein receptor binding domain in BoNTs of other serotypes.

## Materials and Methods

### 1. Ethics Statements

Mice were purchased from Animal Center of Chinese Academy of Sciences and maintained under pathogen-free conditions. All animal experimental procedures were carried out in strict accordance with the guidelines of the Animal Experiment Committee of the International Joint Cancer Institute, and were approved by the Animal Experiment Committee of the International Joint Cancer Institute.

### 2. Holotoxin, Antigens, protein and peptides

BoNT/A, BoNT/B, BoNT/E and BoNT/F were provided by Lanzhou Institute of Biological Products (Lanzhou, China). DNA encoding BoNT/A Hc (residues 868 through 1296), BoNT/B Hc (residues 853 through 1291), BoNT/E Hc (817 through 1255), BoNT/F Hc (847 through 1280), and Syt II-LD fragment (residue 37 through 86) were synthesized and subcloned into a pET expression vector, expressed in E. coli BL21(DE3). The recombinant histidine tagged BoNT/A, B, E, F Hc and Syt II-LD fragment were isolated and purified by nickel affinity gel column chromatography and their molecular weight and purity were verified by gel electrophoresis [31], [32].

Peptides used in this study, P1 (YFCISKWYLKEV), P2 (PISDSDEFYNTI), P3 (IKLKKDSPVGEI), P4 (THPHLPRALMRS), were synthesized by Yeli Bio-Scientific Inc. (Shanghai, China).

### 3. Animals and cell lines

6- to 8-week-old female BALB/c mice were purchased from Animal Center of Chinese Academy of Sciences and maintained under pathogen-free conditions. PC12 (rat adrenal pheochromocytoma cells) was purchased from Cell Bank of Chinese Academy of Science (Shanghai, China). To generate cells that express syt II (syt II), full-length mouse syt II (Genechem. Shanghai, China) was subcloned into pCDNA3.1 (CLONTECH, Mountain View, CA) and transfected into PC12 cells via electroporation. Transfected cells were selected with 1 mg/ml G418, and several independent monoclonal cell lines were established. For experiment in which cells were preloaded with gangliosides (Merk Chemicals, Darmstadt, Germany), cells were grown to 80% confluence followed by incubation in serum-free media plus 250 µg/ml gangliosides. 24 h later, the serum-free/ganglioside media was replaced with complete media, and the cells were incubated with FITC labeled BoNT/B Hc.

### 4. mAb preparation

6-week-old female BALB/c mice were subcutaneously immunized twice at 3-week intervals with 10 ìg of BoNT/B Hc emulsified in Freund's complete or incomplete adjuvant (Sigma-Aldrich, Shanghai, China). Three days after a final immunization with BoNT/B Hc antigen alone, spleen cells from the mice and mouse myeloma NS1 cells (Cell Bank, Chinese Academy of Science, Shanghai, China) were fused and maintained according to the standard procedure [33]. The hybridomas producing anti-BoNT/B antibodies were screened by an indirect enzyme-linked immunosorbent assay (ELISA), using the purified recombinant BoNT/B Hc as a coated antigen. For ascites production, 5×10^6^ hybridoma cells were injected intraperitoneally into BALB/c mice, which had been primed with 0.5 ml pristane (Sigma-Aldrich, Shanghai, China). Ascites fluid was collected, and the mAbs purified, using HiTrap Protein G coupled to Sepharose 4B (Amersham Bioscience, Uppsala, Sweden). mAb isotypes were determined by the Mouse Typer Isotyping Panel Kit (Bio-Rad, Hercules, CA). The binding specificities of the mAbs were determined by western blotting. The binding strength of neutralizing mAbs to the BoNT/B Hc was analyzed by surface plasmon resonance technology using a Biacore T100 instrument (Amersham Bioscience; Uppsala, Sweden) [34].

### 5. Binding specificity and cross-reactivity assay by western blot and ELISA

0.1 µg of purified BoNT/B Hc were mixed with 2× SDS sample buffer (125 mM Tris pH 6.8, 4% SDS, 10% glycerol, 0.006% bromophenol blue, 1.8% ß-mercaptoethanol), boiled and loaded onto 10% polyacrylamide gels. After electrophoresis, the samples were transferred on nitrocellulose sheets, probed with all of these 16 positive binding mAbs respectively (1∶1000), and stained with a goat anti-mouse IgG coupled to horseradish peroxidase (HRP) (BD Bioscience, San Jose, CA) and developed with ECL Plus.

To determine the cross-reactivity of 8E10, Easy Wash 96-well plates (Corning, Corning, NY) were coated at 4°C overnight with 100 µl/well purified BoNT A/B/E/F Hc (at 5 µg/ml) respectively (BSA was set as negative control). After blocked with blocking buffer (5% non fat dry milk in PBS) for 2 hours at room temperature, 1 ng of 8E10 in 100 ul PBS was added and incubated for 2 hour at 37°C. The wells were washed and incubated at 37°C for 1 h with HRP-conjugated goat anti-mouse IgG at a 1∶3000 dilution. After extensive washing, 3, 3, 5, 5-tetramethylbenzidine (TMB) was added (50 ul/well) and incubated for 10 min at room temperature in the dark and the reaction was stopped by the addition of 2 M H2SO4 (50 ul/well). Absorbance of the samples in the plates was read in an automated ELISA microplate reader at 450 nm. For western blot, purified BoNT A/B/E/F Hc were subjected to SDS PAGE, transfer to nitrocellulose membrane and probed with 8E10 as described above.

### 6. Kinetic analysis

BIAcore measurements were performed with the Biacore T200 instrument (GE Healthcare) at 25°C in running buffer (10 mM Tris pH 8.0, 100 mM NaCl, 0.005% surfactant P20, 50 µM NiCl). CM5 chips were coated with BoNT/B Hc. 7 neutralizing mAbs were passed over the chip surface at concentrations ranging from 4.4 nM to 22.2 nM for 240 seconds at a flow rate of 30 ul/min and dissociation was recorded during 60 minutes. The chip was regenerated with 20 µl of 35 mM EDTA at 50 µl/min. Binding kinetics were evaluated using the BiaEvaluation software package (GE Healthcare) using a Langmuir model 1∶1.

To determine the binding abilities of mAb 8E10 and BoNT/A/B/E or F Hc, Protein A was cross-linked to the dextran surface of a CM5 sensor chip. Protein A was immobilized using amine coupling with 1-ethyl-3-[3-dimethylaminopropyl] carbodiimide hydrochloride and N-hydrosuccinimide to a density of 1000–2000 response units (RU). mAb 8E10 was captured to approximately 100 RU. The analyte BoNT/A/B/E or F Hc was passed over the chip surface at concentrations ranging from 4.4 nM to 22.2 nM as indicated for 240 seconds at a flow rate of 30 µl/min and dissociation was recorded during 60 minutes. Binding kinetics were evaluated as described above.

### 7. Competition ELISAs

To identify the binding domain which was blocked by the neutralizing mAbs, BoNT/B Hc was conjugated with horseradish peroxidase (HRP) using HRP Plus Activated Conjugation Kit (Thermo, Rockford, IL) according to the instruction of the kit. Easy Wash 96-well plates (Corning, Corning, NY) were coated at 4°C overnight with 100 µl/well recombinant syt II-LD (at 5 µg/ml) or GT1b (at 10 µg/ml) in PBS, washed with PBS/0.05% Tween-20 (Sigma-Aldrich, Shanghai, China) and then blocked for 1 hour at 37°C with PBS/0.05% Tween-20/5% bovine calf serum/3% goat serum (Sigma-Aldrich, Shanghai, China). HRP-conjugated BoNT/B Hc was pre-incubated with neutralizing mAbs at molar ratio of 1∶100 for 1 hour at 37°C before added into coated plate. 3, 3, 5, 5-tetramethylbenzidine (TMB) was used as a substrate (Sigma-Aldrich, Shanghai, China) to detect bound BoNT/B Hc.

To determine the binding epitope of the mAbs were overlapping or not, competition was tested by the competition-ELISA [35] with some modifications. Mixtures of constant quantities of the HRP-mAbs (8D1, 8E10, 1F4, 5G10, 2H12, 2B2 and 2F4) conjugate in the optimal working dilutions with dilutions of the competing non-labelled mAb were incubated on BoNT/B Hc coated plates for 1 hour at 37°C. Follow a final wash cycle, 50 ul of substrate and stopping solution were added and absorbance values were determined as previously described. A control without competing antibody (PBS) and a control containing the same mAb (self-competition) were included in the test. Results were expressed as percent binding of HRP-conjugated mAb. The amount of binding obtained in the absence of unlabeled antibody was set at 100% for each HRP-conjugated mAb.

### 8. Epitope mapping by biopanning

Anti-BoNT/B antibodies (8E10, 5G10 and 2F4) were separately immobilized on 96-well plate. Phages (1.5×10^11^ pfus) from Ph.D.-12 Phage Display Peptide Library (New England Biolabs, Beijing, China) were pre-absorbed by immobilized mouse IgG. The phages, diluted in Tris-buffered saline, were incubated at 4°C for 1 h with immobilized mAbs. The wells were washed for five times with TBST (0.1% Tween 20/Tris-buffered saline). Then, the phages that bound with mAbs were amplified by direct infection with E. coli ER2738. The amplified phages were purified by precipitation with 20% PEG8000, 2.5 M NaCl and used in the next cycle. Three rounds of selection were routinely performed.

The indirect binding assay to test the reactivity of anti-BoNT/B Hc mAbs with synthetic peptides was performed in 96-well plates as described previously [36] with minor modifications. The plates were coated with KLH-peptide 1, 2, 3 respectively. Following 2 washings and blockade of free protein-binding sites, mAbs were added to each well respectively. Mabs binding to peptide were detected by sequential addition of an appropriate dilution of HRP-conjugated goat anti-mouse IgG. To confirm the specific of the epitopes identified, anti-BoNT/B mAbs were incubated with synthetic peptides (P1, P2, P3) respectively, and then the mixture was respectively added to plates precoated with BoNT/B Hc. After 2 h incubation, the wells were washed and detected with an appropriate dilution of HRP-conjugated secondary antibody. After the addition of TMB and stop solution, absorbance was read at 450 nm by a microplate reader.

### 9. Flow cytometry assay

The inhibition of mAbs for cell binding activity of BoNT/B was examined by performing flow cytometry [37]. BoNT/B Hc was labeled with the FITC labeling kit according to the instruction (Pierce Biotechnology, Rockford, IL).The single or pairs of mAbs were mixed with FITC-BoNT/B Hc at 37°C for 1 h. Ganglioside-treated or Syt II transfected (Syt II+) or double positive PC12 cells were labeled by overnight incubation with the mixture at 4°C and washed with cold PBS, and examined by flow cytometry (BD Biosciences, San Jose, CA). The PC12 cells alone and FITC-labeled Hc fragment of BoNT/B were used as systemic control and negative control, respectively.

### 10. Confocal microscopy

FITC labeled BoNT/B Hc (1 µg) was incubated with control medium, monoclonal antibody (100 µg) or pairs of mAbs (50 µg of each Ab) for one hour at room temperature. Sub-confluent Ganglioside-treated SytII^+^-PC12 cells, plated on glass cover slips, were incubated with the BoNT/B Hc-mAbs mixtures at 4°C for one hour and then for 2 hours at 37°C. Cells were then washed with PBS for 15 minutes, fixed with 4% paraformaldehyde for 15 mins, and washed again before mounting. Confocal laser scanning was performed on a Olympus Fluoview 500 system using the 40×objective, and Fluoview software was used for image analysis.

### 11. Neutralization of BoNT/B activity *in vivo*

For the initial screening of the positive hybridoma that produced the neutralizing antibody, 0.5 ml of culture supernatant from hybridomas grown in a 24-well tissue culture flask for 3–5 days, was pre-incubated with 4 LD50 of BoNT/B for 1 h at room temperature, and the reaction mixtures intraperitoneally (i.p.) injected to each of six mice. More than four survivals in four days were considered as positive. In order to define the activity of the positive neutralizing mAbs and synergistic effect of pairs of mAbs, 100 µg of the positive neutralizing mAbs were added to the indicated number of mouse LD50 of BoNT/B neurotoxin in a total volume of 0.5 ml of gelatin phosphate buffer and incubated at RT for 60 min. For pairs of mAbs, 50 µg of each mAb was added. The mixture was then injected i.p. into female BALB/c mice (16–22 g). Mice were studied in groups of six and were observed at least daily. The morbidity and mortality of mice were determined over 30 days. For the cross-protection assays, 100 µg of 8E10 was pre-incubated with 20 LD50s of BoNT/A, B, E, F for 1 hour respectively. The mixtures were injected into mice, and the final death tally was determined 4 days after injection.

## Supporting Information

Figure S1 The effect of BoNT/B-specific antibodies on binding of BoNT/B Hc with ganglioside-treated Syt II+PC12 cells. Ganglioside-treated Syt II+PC12 cells were cultured with FITC-labeled BoNT/B Hc (green), with or without mAbs as indicated and visualized by confocal microscopy.
(DOC)

Figure S2 Binding affinity of mAb 8E10 and BoNT A/B/E/F Hc. The 8E10 was immobilized on the surface of a Protein A cross-linked CM5 chip. Purified BoNT/A/B/E/F Hc at concentrations of 22.2 nM, 14.8 nM, 9.9 nM, 6.6 nM, 4.4 nM were directly injected over the target and the reference surface. The signal obtained from the reference surface was subtracted to avoid the binding of non-specificity.
(DOC)
