# Supplementary material for: Potent Neutralization of Botulinum Neurotoxin/B by Synergistic Action of Antibodies Recognizing Protein and Ganglioside Receptor Binding Domain
Source: PLoS One. 2012 Aug 29;7(8):e43845. doi: 10.1371/journal.pone.0043845 (PMC3430616; doi:10.1371/journal.pone.0043845)
Supplement: Figure S1 — The effect of BoNT/B-specific antibodies on binding of BoNT/B Hc with ganglioside-treated Syt II+PC12 cells. Ganglioside-treated Syt II+PC12 cells were cultured with FITC-labeled BoNT/B Hc (green), with or without mAbs as indicated and visualized by confocal microscopy. (DOC) [file pone.0043845.s001.doc]

**
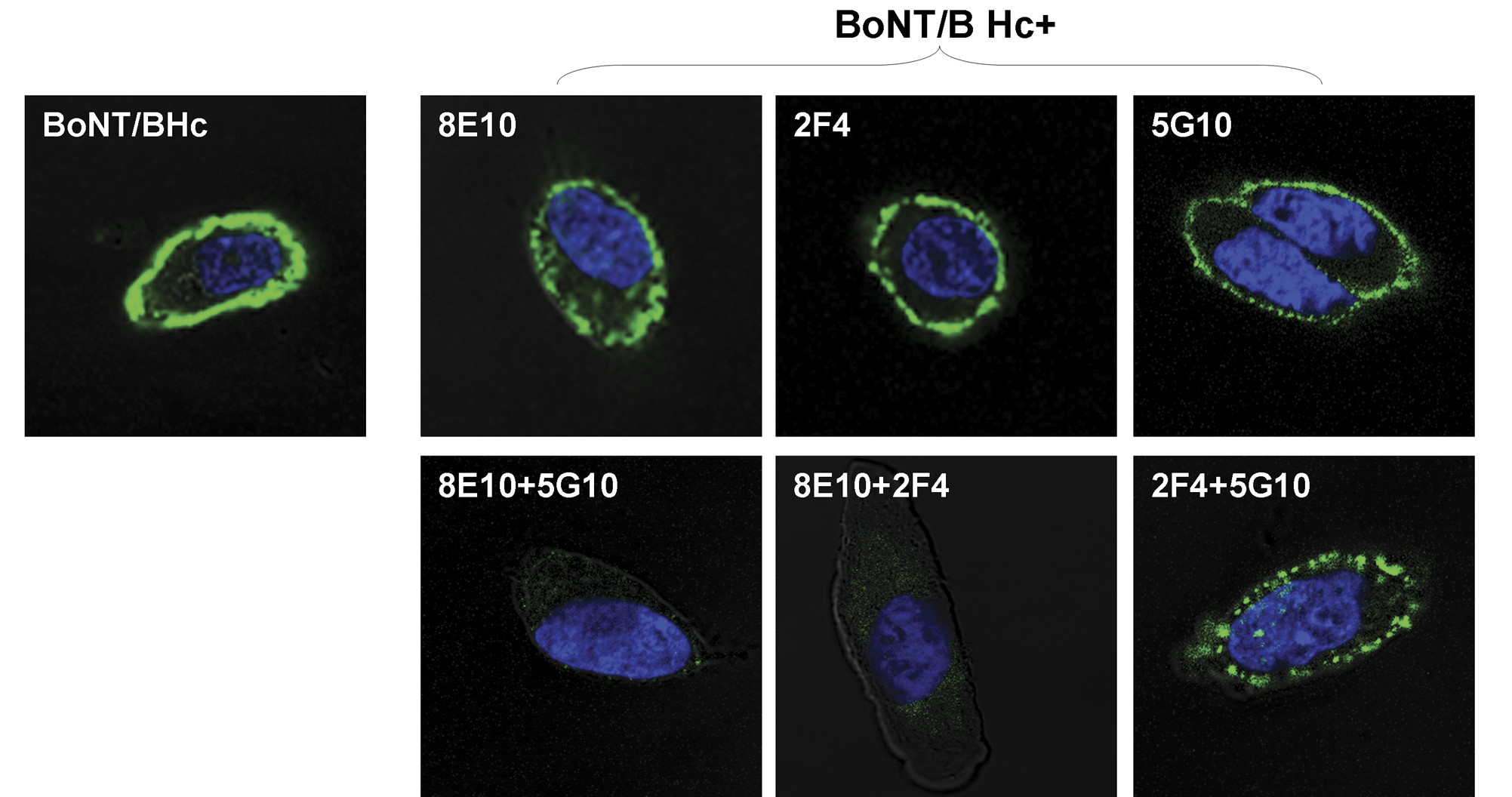
**

**Figure S1 The effect of BoNT/B-specific antibodies on binding of BoNT/B Hc with ganglioside-treated Syt II+PC12 cells.**

Ganglioside-treated Syt II+PC12 cells were cultured with FITC-labeled BoNT/B Hc (green), with or without mAbs as indicated and visualized by confocal microscopy.
