# Supplementary material for: Potent Neutralization of Botulinum Neurotoxin/B by Synergistic Action of Antibodies Recognizing Protein and Ganglioside Receptor Binding Domain
Source: PLoS One. 2012 Aug 29;7(8):e43845. doi: 10.1371/journal.pone.0043845 (PMC3430616; doi:10.1371/journal.pone.0043845)
Supplement: Figure S2 — Binding affinity of mAb 8E10 and BoNT A/B/E/F Hc. The 8E10 was immobilized on the surface of a Protein A cross-linked CM5 chip. Purified BoNT/A/B/E/F Hc at concentrations of 22.2 nM, 14.8 nM, 9.9 nM, 6.6 nM, 4.4 nM were directly injected over the target and the reference surface. The signal obtained from the reference surface was subtracted to avoid the binding of non-specificity. (DOC) [file pone.0043845.s002.doc]

**
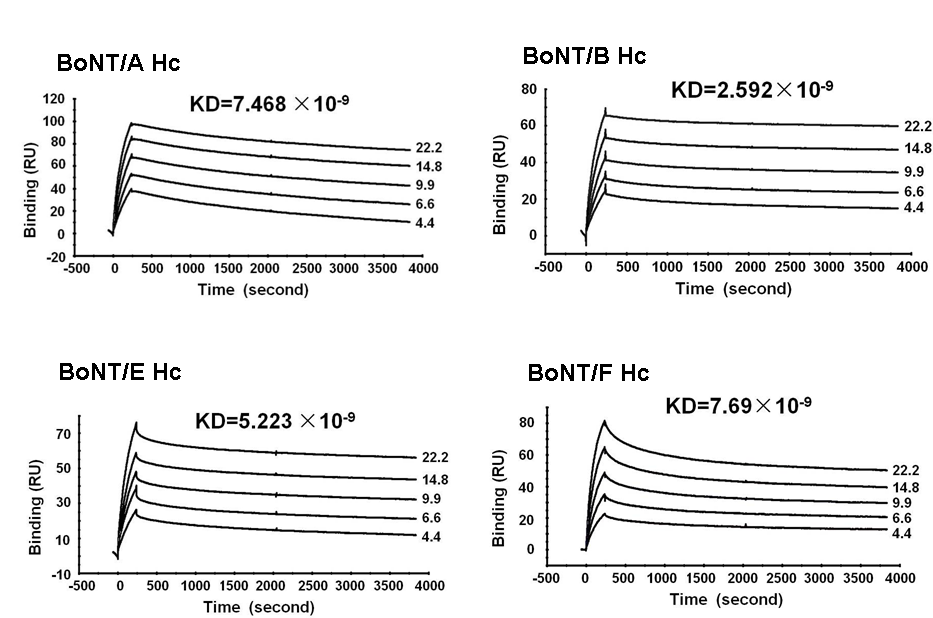
**

**Figure S2 Binding affinity of mAb 8E10 and BoNT A/B/E/F Hc**

The 8E10 was immobilized on the surface of a Protein A cross-linked CM5 chip. Purified BoNT/A/B/E/F Hc at concentrations of 22.2nM、14.8nM、 9.9nM、6.6nM、4.4nM were directly injected over the target and the reference surface. The signal obtained from the reference surface was subtracted to avoid the binding of non-specificity.
